# Frequency of adverse perinatal outcomes in patients with pregnancy related acute renal (kidney) injury in a tertiary care hospital

**DOI:** 10.12669/pjms.40.10.9228

**Published:** 2024-11

**Authors:** Noor Mohammad, Qudsia Qazi, Nazia Liaqat

**Affiliations:** 1 Noor Mohammad, MBBS, MCPS(Medicine), FCPS Nephrology Associate Professor, Department of Nephrology, Medical Teaching Institute, Lady Reading Hospital, Peshawar, Pakistan; 2 Qudsia Qazi, MBBS, FCPS (Obstetrics & Gynecology) Associate Professor, Department of Gynecology, Medical Teaching Institute, Lady Reading Hospital, Peshawar, Pakistan; 3 Nazia Liaqat, MBBS, FCPS (Obstetrics & Gynecology) Associate Professor, Department of Gynecology, Medical Teaching Institute, Lady Reading Hospital, Peshawar, Pakistan

**Keywords:** Adverse perinatal outcome, Stillbirth, Pr-AKI, Persistent renal failure

## Abstract

**Background & Objective:**

Pregnancy related acute kidney injury (Pr-AKI) is coupled with adverse feto-maternal outcomes. Adverse perinatal outcome comprising of still births, intrauterine growth retardation, neonatal deaths are indicators of severity of underlying obstetrical conditions ending on Pr-AKI. These perinatal outcomes can also serve as potential predictors for long term outcomes of Pr-AKI. Our study aimed to determine frequencies of adverse perinatal outcomes and to evaluate adverse perinatal outcome as predictor for persistent renal injury in patients with pregnancy induced acute renal injury (Pr-AKI).

**Method:**

A descriptive case series including 100 women with Pr-AKI, was conducted in a tertiary care hospital of Peshawar from 1st August 2021 to 31st July 2022. Included patients were followed for three months period, from their date of delivery. Adverse perinatal outcome included stillbirths, and early neonatal deaths.

**Results:**

The mean age of study sample was 29.20±6.40. The pre dominant etiology for Pr-AKI was primary postpartum hemorrhage, occurring in 52% women. Still births were seen in 48% cases, while early neonatal deaths were seen in 7% cases. Adverse perinatal outcome had statistically significant association with persistent renal failure (p-0.01). Other factors having statistically significant association with persistent renal injury (PRF) were multiparity and cesarean births. (p<0.05) Association of adverse perinatal outcome with persistent renal injury persisted on multivariate logistic regression. a OR 6.14; CI 1.15-32.29, p-0.033.

**Conclusion:**

Almost half of the cases with Pr-AKI have still births. Still birth in patients with Pr-AKI is associated with persistent renal injury at 12 weeks follow up period.

## INTRODUCTION

Obstetrical acute kidney injury occurs as a result of conditions or complications specific to pregnancy. Conditions in pregnancy that are known to be associated with renal injury include hypertensive disorders of pregnancy, HELLP syndrome, abruptio placenta, acute fatty liver of pregnancy. While complications like,hemorrhage and sepsis in pregnancy and puerperium, if not managed timely and appropriately,can lead to renal injury.^1^,^2^

The incidence of Pr-AKI is reported to be 2.0% in a meta-analysis of 31 studies.^3^ There has been significant drop in the incidence of obstetrically induced renal injury in developed countries. In the developing countries Pr-AKI is a significant health problem because of lack of access to health care, delay in diagnosis and referrals of women with potential high-risk conditions in pregnancy. AKI in pregnancy and puerperium is found to be associated with substantial risks of maternal mortality and morbidity. Pregnancy related AKI is also recognized to progress to renal failure, chronic kidney diseases and end stage renal failure.^4^

Apart from maternal implications, it is also related to adverse perinatal outcome. Adverse perinatal outcome includes intra uterine growth retardation, intra uterine fetal deaths, early neonatal deaths, prolonged neonatal intensive care admissions. Antepartum hemorrhage, hypertensive disorders in pregnancy and sepsis are the leading causes of adverse perinatal outcomes as well as of, maternal mortality and morbidity including Pr-AKI in developing countries.^5^,^6^

The current study aimed to determine frequencies of various adverse perinatal outcomes in women with pregnancy induced acute renal injury and to evaluate adverse perinatal outcome as predictor for persistent renal injury in these women. This study will open window for larger studies on this aspect of AKI in pregnancy, which will help in counselling of women regarding their risks of persistent renal failure on one hand and closer surveillance and monitoring of high-risk women (those with poor perinatal outcome).

## METHODS

This was a descriptive case series. It was conducted at Nephrology unit in collaboration with department of OBGYN of Lady Reading hospital, Peshawar from 1st August 2021 to 31st July 2022. Sample size of 100 was calculated via online sample size calculator, by taking confidence level as 95% and proportion of persistent renal injury in women with pregnancy induced kidney injury as 6.7%.^7^

### Ethical approval:

The hospital ethical review board approved the study. (Ref No. 188/LRH/MTI, dated: July 12, 2021). A total of hundred women fulfilling the eligibility criterion were recruited into the study. Non probability consecutive sampling technique was utilized. Informed consents were acquired from all women at enrollment.

### Study eligibility criterion:

Patients with pregnancy related acute renal injury, at term or within first week of delivery, were enrolled into this study. Women with pre -existing kidney diseases, autoimmune diseases, those having renal calculi, or having small echogenic kidneys bilaterally and with lethal fetal congenital anomalies were excluded. AKIN criterion was utilized, which needs any of the following for diagnosis of renal injury:

- increase in serum creatinine by 0.3 mg/dl or more in 48 hours,
- rise in serum creatinine to 1.5 times or more of baseline values within the last seven days,
- urine output of less than 0.5 ml/ kg/hour for six hours.^8^
- Renal injury was considered to be persistent in women, who still needed dialysis at follow up at 12 weeks postpartum. Adverse perinatal outcome included stillbirths, and early neonatal deaths.

A pre designed proforma was used to collect patient’s details. Information including age of woman, parity, period of gestation at delivery, presence of regular antenatal care, presence of anemia, hypertension, diabetes, infection, mode of delivery, place of delivery, underlying obstetrical cause leading to acute renal injury, history of blood transfusions and perinatal outcome, were recorded. All the included women were followed till 12 weeks postnatal period. Data was analyzed on SPSS version 25.0. Frequencies and percentages for categorical variables and mean, and standard deviations for numerical variables, were computed. Association between categorical variables was checked via Chi square test. Multivariate logistic regression analysis was carried out for variables showing significant association with persistent renal failure to get adjusted odd ratios.

## RESULTS

The mean age of sample of 100 women was 29.20±6.40 with a range of 18-44 years. There were 89% multiparas and 11% primiparous women in the study. Postpartum hemorrhage was seen as cause of Pr AKI in 52% cases, sepsis in 18% and hypertensive disorders of pregnancy in 11% cases. Regarding mode of deliveries, majority of patients i.e., 96% women delivered in hospitals. Only four percent women had history of home delivery. Majority i.e., 78% had vaginal delivery and 22% had cesarean section for obstetrical reasons.

Thirteen patients were lost to follow up. Majority of patients i.e., 66% had complete recovery at 12 weeks postpartum period. In this study, the frequency of persistent renal injury (PRI) in women with Pr-AKI was 14%. Regarding fetal outcomes, the still births were seen in 48% cases, early neonatal deaths were seen in 7% cases and, alive healthy babies at the end of study period were 45%. Adverse perinatal outcomes had statistically significant association with persistent renal injury at follow up. (p-0.01) (Table-I). Cesarean births were also noted to have statistically significant association with PRF at follow up visits. As 36.8% (7/19) women with cesarean birth had PRF as compared to 10.44% (7/67) women with normal birth. (p-0.01). Underlying obstetrical causes of Pr-AKI and place of delivery did not show statistical significance with regard to occurrence of PRF. (P >0.05). The association of history of hemodialysis and massive blood transfusion with PRF was also statistically insignificant. (P>0.05)

**Table-I:** Association of adverse perinatal outcome with persistent renal failure in patients with Pr-AKI.

Perinatal outcomes	Persistent renal failure cases			Total	p-values

	No	Yes	Loss to follow ups		
Alive	37	02	06	45	0.01
Still births	33	12	03	48	
Neonatal deaths	03	00	04	07	
Columns total	73	14	13	100	

Univariate and multivariate analysis were carried out to determine strength of significant associations and to control for potential confounding effects of variables. Multiparity, mode of delivery, history of massive blood transfusion at delivery, history of hemodialysis and adverse perinatal outcome were included in logistic regression model.

Adjusted odd ratio was computed for significant associations after controlling for confounders like parity, place of delivery and mode of delivery, history of blood transfusion and hemodialysis via multivariate logistic regression analysis. The adjusted odd ratio for association of still birth and persistent renal failure was found out to be 6.14; CI 1.15-32.29, p-0.033 (Table-II).

**Table-II:** Multivariate logistic regression for association of adverse perinatal outcome and persistent renal failure.

Perinatal outcomes	Persistent renal failure		p-value	Univariate analysis (Model 1)	Multivariate analysis	p-value	95%Cconfidence Interval

	No	Yes					

				Crude OR	Adjusted OR		
***ALIVE***	***Reference***						
1.Still Births	33	12	0.017	6.93	6.14	0.033	1.15-32.29
***Vaginal delivery***	***Reference***						
2.Cesarean section	12	07	0.01	5.08	1.99	0.333	0.50-7.99
***Primiparity***	***Reference***						
3. Multiparity	65	13	0.03	1.2	1.20	0.88	0.16-12.89
***Hemodialysis***	***Reference***						
4.No need for Hemodialysis	21	01	0.08	0.17	0.20	0.15	0.02-1.83
***No massive blood transfusion***	***Reference***						
5.Massive blood transfusion	37	11	0.06	2.57	1.21	0.79	0.29-4.9

## DISCUSSION

In this study the frequency of adverse perinatal outcome was 45% for still births and 7% for early neonatal deaths. Adverse perinatal outcome was found to have significant association for persistence of renal injury at 12 weeks postpartum time period. In a study done by Rekha Sachan and colleagues in India, high rates of still births similar to current study findings were reported in women with Pr-AKI. In that study the rates of still births/IUFDs were 53.8% for Stage II AKI and 37.7% for Stage III AKI, compared to none for stage-I. In their study, they also had a fairly good rate of institutional deliveries i.e 93.3%.^9^ In the current study, the rate of hospital deliveries was 96%.

This finding of nearly all women with Pr-AKI delivering in hospitals, could be due to the reason that many women with antepartum hemorrhage, sepsis and hypertensive disorders of pregnancy ultimately present to tertiary care hospitals. But these cases are often too late to seek medical advice and hence, by the time they make it to health facilities, many of the complications have already occurred or are difficult to prevent. Another finding that emerged during the current study was association of cesarean births with PRF.This may be due to the reason that in many high risk obstetrical conditions or complications, cesarean sections are preferred to expedite births in the index hospital. In contrast to current study the complete recovery rates in the same Indian study were low i.e 27.3% with progression to chronic kidney disease in 3.3%.Remaining cases either ended in mortality or partial renal recovery. They did not study association of perinatal outcome with persistent renal injury.

In another study of 354 patients at risk of pregnancy related Pr- AKI, the perinatal mortality rate was 15.4% with AKI. This was not statistically significant when compared with high-risk women without AKI. In the same study, the complete renal recovery rate was 84.6% and no women with AKI needed dialysis.^10^ A systematic review including14 studies reported a wide range of perinatal mortality i.e.1.5-60.5%, among patients with pregnancy related Pr- AKI.^11^

This reflects great heterogenicity in the studies in terms of reported perinatal outcome. It may also be due to the fact that the etiology of perinatal mortality is often multifactorial. It depends on factors like severity of underlying condition, timeliness and appropriateness of treatments of maternal conditions and, availability of neonatal care services. In another study conducted in India, the perinatal death rates were 23.5%, primarily resulting from intrauterine deaths in 17.5% cases and pre-maturity in 6% cases.^7^

The current study findings are close to a study conducted by Gopalakrishnan back in 2014, who reported live birth rate of 42%.^12^ Perinatal mortality was reported to be 50%, and dialysis dependency as 3.1% in another study done by Adejumo OA.^13^ All the above studies referred to, are from the developing world. The high rates of adverse perinatal outcome reported in these studies, signifying the severity of pregnancy specific disorders reflect the burden of complications.

In a multi-centre study done in South Africa, the risk of still birth was higher (relative risk 2.2;95% CI:1.8-2.8) in patients with AKI in comparison to those who did not have AKI. The risk of perinatal mortality was also high. (89/240;37.1%). The strongest predictor of AKI in that study was history of hypertension in previous pregnancy.^14^

In another study the incidence of acute renal injury was 42.86% among women with severe pre-eclampsia.^15^ The findings of current study are very close to another study conducted in Pakistan which also reported postpartum haemorrhage to be the predominant cause of renal injury in pregnancy.^16^ The rate of chronic renal injury in that study was 13.9%, very much close to 14% found out by this study. The current study was a prospective observational study on an understudied aspect of Pr-AKI.

### Limitations:

This was a single centred study representing experience with limited number of cases and also there was no control group.

## CONCLUSION

Adverse perinatal outcomes including still births and early neonatal deaths are fairly common in patients with pregnancy related acute renal injury and these adverse perinatal outcomes have strong association with persistence of renal injury at 12 weeks postpartum period.

### Authors Contribution:

**NMK:** Conceptualization, data collection, editing of manuscript.

**QQ:** Design, editing and finalization of manuscript.

**NL:** Data Analysis, literature search, manuscript writing.

All the three authors are responsible for originality and integrity of the research.
